# Artificial Intelligence Algorithm-Based High-Resolution Computed Tomography Image in the Treatment of Children with Bronchiolitis Obliterans by Traditional Chinese Medicine Method of Resolving Phlegm and Removing Blood Stasis

**DOI:** 10.1155/2022/8952791

**Published:** 2022-05-27

**Authors:** Xiaoning Shi, Qing Zhou

**Affiliations:** ^1^Child Health Section, Hunan Province Directly Affiliated TCM Hospital, Zhuzhou, Hunan 412000, China; ^2^Department of Orthopedics and Traumatology, Zhuzhou 331 Hospital, Zhuzhou, Hunan 412000, China

## Abstract

This research was aimed to explore the application of high-resolution computed tomography (HRCT) based on intelligent iterative reconstruction technique in the early diagnosis and treatment of bronchiolitis obliterans (BO) in children and to explore the efficacy of traditional Chinese medicine (TCM) in resolving phlegm and removing blood stasis. Sixty pediatric patients with BO were selected as the study subjects and diagnosed by HRCT scanning, and the scanned images were processed by iterative reconstruction technique. The patients were treated with TCM therapy of resolving phlegm and removing blood stasis alone (group A), HRCT-guided TCM therapy of resolving phlegm and removing blood stasis (group B), and iterative reconstruction HRCT-guided TCM therapy of resolving phlegm and removing blood stasis (group C). The results showed that the lung HRCT image after iterative reconstruction was closer to the original image than that after filtered back projection reconstruction, and the edge of the image after filtered back projection reconstruction was more blurred and the noise was higher. The image obtained by iterative reconstruction technique was smoother and clearer, and the image stability after iterative reconstruction was higher. The treatment results showed that the proportion of moderate and severe obstruction in group C was 5.18%, which was significantly lower than that in group A (18.75%) and group B (11.29%), and group B was significantly lower than that in group A (18.75%) (*P* < 0.05). The proportion of clinical effect in group C after treatment was 70.18%, significantly higher than that in group A (55.5%) and group B (63.34%), and that in group B was significantly higher than that in group A (55.5%) (*P* < 0.05). In summary, the lung HRCT after iterative reconstruction can more clearly and intuitively show the lesion site, which has a key role in guiding the early diagnosis and treatment planning of BO; the HRCT image based on iterative reconstruction technique combined with TCM treatment of removing blood stasis and resolving phlegm has a better therapeutic effect on children, with a high application value.

## 1. Introduction

Bronchiolitis obliterans (BO), also known as constrictive bronchiolitis, is a chronic airflow obstruction syndrome associated with inflammatory injury of small airways and belongs to a rare, fatal, and irreversible obstructive pulmonary disease that manifests as bronchiolar narrowing or obstruction due to inflammation or fibrosis [1, 2]. Since BO is usually caused by secondary respiratory tract infection, of which the more common is viral infection, and adenovirus is predominant, the disease is also used to specifically refer to a severe subtype of bronchiolitis in children caused by adenovirus or is considered to be a pulmonary manifestation in the chronic phase of transplant rejection [3, 4]. The main clinical characteristics of the disease are recurrent or persistent shortness of breath, wheezing or coughing, poor motor ability, and fine rales and wheezing in the lungs [5, 6]. Relevant studies indicated that the target of the pathogen causing bronchiolitis obliterans is respiratory ciliary cells. Due to the immune response mediated, inflammatory reaction and fibrosis occur in the repair process of epithelial cells, resulting in bronchiolitis obliterans [7, 8].

The treatment of bronchiolitis obliterans is generally treated with anti-inflammatory therapy so that the occlusion of bronchiolitis can be relieved. For bronchiolitis caused by bacterial infection, antibiotics should be used for treatment [9]. In addition, according to clinical experience, the combination of TCM treatment can often well improve the symptoms of children, improve the symptoms of cough, wheezing, and shortness of breath, and reduce the frequency of recurrent respiratory tract infections. TCM believes that bronchitis belongs to the category of lung or asthma syndrome [10, 11].

High-resolution computed tomography (HRCT) is widely used in clinical diagnosis of pulmonary nodules and nodules with special imaging [12, 13]. HRCT can clearly display fine structures, such as pulmonary lobules, airways, blood vessels, and interstitium, and does not require contrast enhancement during scanning [14]. HRCT is mainly used in the diagnosis of unexplained acute or chronic dyspnea, hemoptysis, lymphangitis cancer, emphysema, idiopathic interstitial fibrosis, and other diseases [15, 16]. Compared with conventional computed tomography (CT), HRCT has the advantage of high resolution, but its wide application in clinical diagnosis and treatment has certain limitations due to its high radiation dose [17, 18]. Iterative reconstruction algorithm (ART) refers to reconstructing images by solving linear equations. Starting from a hypothetical initial image, the method of gradual approximation is adopted. The theoretical projection value is constantly compared with the actual measured projection value and updated iteratively until the optimal solution is finally obtained. It was first used in positron emission tomography imaging, which can obtain high-quality images under the condition of low radiation dose [19, 20].

Therefore, a foreign study proposed the concept of low radiation dose scanning; however, reducing the radiation dose will increase the noise of the image to some extent. In order to solve the problem of increased image noise caused by the use of low radiation dose scanning, an iterative reconstruction algorithm was used for the reconstruction of HRCT images of the lungs so as to study the therapeutic effect of HRCT images based on iterative reconstruction algorithm combined with TCM for resolving phlegm and removing blood stasis on BO.

## 2. Materials and Methods

### 2.1. General Data and Grouping

Sixty patients with pediatric (32 males and 28 females) with BO admitted to hospital from September 2018 to November 2020 were randomly divided into group A (TCM treatment of resolving phlegm and removing blood stasis alone), group B (HRCT-guided TCM treatment of resolving phlegm and removing blood stasis), and group C (iterative reconstruction of HRCT-guided TCM treatment of resolving phlegm and removing blood stasis), with 20 patients in each group. The study was approved by the ethics committee of hospital. The patients and their families understood the study content and methods and signed the corresponding informed consent form.

TCM syndrome diagnosis criteria: cough weakness, increased sputum volume, fatigue, and sluggishness; tongue purple dark or spotted, thin and white coating.

Inclusion criteria: (i) children who met the clinical diagnostic criteria of bronchiolitis obliterans in children and were in stable stage; (ii) TCM syndrome differentiation belongs to deficiency of spleen and lung and internal obstruction of phlegm and blood stasis; and (iii) the age ranges from 4 to 15.

Exclusion criteria: (i) patients with diseases of other systems or organs; (ii) children with bronchial asthma, foreign body inhalation, and congenital bronchopulmonary dysplasia; and (iii) patients with severe liver and kidney insufficiency and primary diseases.

### 2.2. HRCT Scanning of Lung

During flat scan, the child was supine, arms up. It first scanned the positioning film and then determined the scanning range on the positioning film. HRCT scanning was employed. The scanning was performed in the transverse position, usually from the tip of the lung to the bottom of the lung, with layer thickness of 8–10 mm and layer spacing of 10 mm. Scanning was performed after deep inspirations and after breath-holding or after calm breathing. The scanning time was generally 0.7–3 s. HRCT scan: large matrix (512 × 512), thin layer (1∼2 mm), and small field of view (15∼30 cm in two lung scanning fields, 15∼20 cm in the first). Image reconstruction was performed using iterative reconstruction technology; scanning field of view (FOV): 25∼35; scan time <1 s.

### 2.3. Treatment of BO

Patients were treated with resolving phlegm and removing blood stasis decoction orally; the prescription composition was as follows: 15 g Radix Astragali, 10 g Radix Codonopsis, 10 g Poria, 10 g Rhizoma Dioscoreae, 10 g Pericarpium Citri Reticulatae, 9 g processed Pinellia Tuber, 10 g Rhizoma Dilong, 10 g Rhizoma Zelan, 6 g unprocessed Radix Glycyrrhizae, and so on. The specific dosage was added and subtracted according to the syndrome. The patients in group B and group C were diagnosed by HRCT and HRCT after iterative reconstruction, and the treatment plan was formulated according to the diagnosis results: 1 month as a course of treatment and continuous treatment for more than 5 courses.

### 2.4. HRCT Image Reconstruction

Image reconstruction algorithms can be roughly divided into transform method and series expansion method; the most used series expansion method is iterative reconstruction. Algebraic reconstruction method, simultaneous iterative reconstruction method, and maximum entropy method are widely used in iterative reconstruction algorithm [21]. Mathematical principle of image reconstruction: the radon transform [*Rf*](*L*, *θ*) of image function *f*(*r*,*ϕ*) is a line integral along the line *L*. It is the sum of the attenuation coefficients of each body element on the ray path, namely, the measured projection data. The essence of image reconstruction problem can be summarized as follows: according to projection data *P*(*L*,*θ*) (equation), the function image *f*(*r*,*ϕ*) can be obtained as follows.

(1) $$\begin{array}{c} \left[Rf\right]\left(L,\theta \right)=\int_{-\infty }^{\infty }f\left(r,\phi \right)\text{d}z,\\ \int_{-\infty }^{\infty }f\left(r,\phi \right)\text{d}z=P\left(L,\theta \right). \end{array}$$

**Figure 1:**
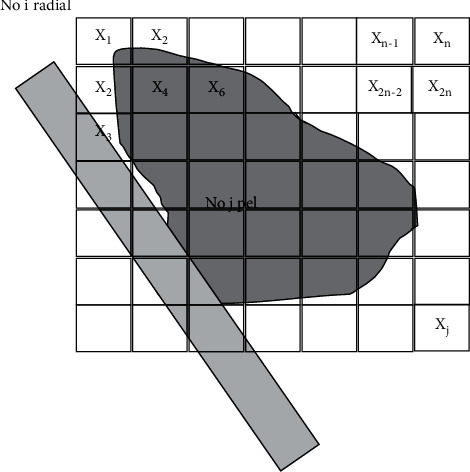
Basic idea of iterative reconstruction algorithm.

The essence of algebraic reconstruction method is to solve the discrete projection equation approximately by iterative method, and the deviation is continuously corrected until satisfactory results are obtained. The algebraic reconstruction method is mathematically to solve a certain mathematical problem (to find a vector that satisfies a given linear inequality), and its basic idea is illustrated in Figure 1.

In Figure 1, the pixel value (gray scale or density) of pixel *j* is represented with *x*_*j*_, and the overlapping area of ray *i* and pixel *j* is the intersection area of the shaded part in the figure, and its area (*R*) ratio to pixel *δ*^2^ is as follows.

(2) $$\begin{array}{c} R_{ij}=\frac{S_{\text{dashed area}}}{\delta^{2}}. \end{array}$$

The ray projection contribution (*T*) of pixel *j* to ray *i* is shown as follows.

(3) $$\begin{array}{c} T_{ij}=R_{ji}X_{j}. \end{array}$$

Ray *j* also passes through other pixels and its total ray projection is as follows.

(4) $$\begin{array}{c} P_{ij}=\sum_{1}^{N}P_{ij},\\ =\sum_{1}^{N}R_{ij}X_{j}. \end{array}$$

Equation (4) can be expressed by the following matrix.

(5) $$\begin{array}{c} P=R_{X}, \end{array}$$

(6) $$\begin{array}{c} P={\left[P_{1},P_{2},\ldots ,P_{j}\right]}^{T}, \end{array}$$

(7) $$\begin{array}{c} X={\left[X_{1},X_{2},\ldots ,X_{i}\right]}^{T}, \end{array}$$

(8) $$\begin{array}{c} R=\left[\begin{array}{cccccc} r_{11} & r_{12} & \cdot & \cdot & \cdot & r_{1j}\\ r_{21} & r_{22} & \cdot & \cdot & \cdot & r_{2j}\\ \cdot & \cdot & \cdot & \cdot & \cdot & \cdot \\ \cdot & \cdot & \cdot & \cdot & \cdot & \cdot \\ \cdot & \cdot & \cdot & \cdot & \cdot & \cdot \\ r_{i1} & r_{i2} & \cdot & \cdot & \cdot & r_{ij} \end{array}\right]. \end{array}$$

*P* is a *j*-dimensional vector (measurement vector); *x* is an *i*-dimensional vector, called image; and *R* is a *ij*-dimensional matrix (projection matrix). According to the measured *P* and the known matrix *R* (*R* can be obtained when the pixel arrangement and the geometric structure of ray are specified), *X* is obtained. Giving the actual situation and inevitable measurement error, (5) can be modified as follows.

(9) $$\begin{array}{c} P=R_{X}+e, \end{array}$$

where *e* is the error vector. An image vector $\hat{X}$ is selected to minimize the objective function $\phi_{1}\left\{\hat{x}\right\}$ (called the main criterion); if there are more than one *x*-value, one of which $\hat{x}$ is selected to minimize the other objective function $\phi_{2}\left\{\hat{x}\right\}$ (called the auxiliary criterion). $\phi_{1}\left\{\hat{x}\right\}$ and $\phi_{2}\left\{\hat{x}\right\}$ are the best criteria by used images. The commonly used optimal criteria include least squares criterion, maximum uniformity criterion, smoothing criterion, and maximum entropy criterion.

The least squares criterion: the following functions are minimized to solve $\hat{x}$. The physical meaning of this criterion is to select $\hat{x}$ to minimize the sum of squares of errors between the generated rays and the measured values.

(10) $$\begin{array}{c} \phi_{1}\left(x\right)=\sum_{i=1}^{I}\left(P_{i}-\sum_{j=1}^{J}r_{ij}{\hat{X}}_{j}\right),\\ \sum_{i=1}^{I}\left(P_{i}-\sum_{j=1}^{J}r_{ij}{\hat{X}}_{j}\right)={\left\|P-\text{R}\hat{X}\right\|}^{2},\\ {\left\|P-R\hat{x}\right\|}^{2}={\left(P-R\hat{x}\right)}^{T}\left(P-R\hat{x}\right). \end{array}$$

Maximum uniformity criterion and smoothness criterion are as follows.

(11) $$\begin{array}{c} \phi_{2}\left(x\right)=\phi_{21}\left(x\right)+\overset{˜}{\phi_{22}}\left(x\right)\\ =X^{T}\text{BX}+X^{T}X\\ =X^{T}\left(B+i\right)X. \end{array}$$

The physical meaning of (11) is the local uniformity between each pixel and its adjacent pixel and the uniformity of the whole image. If the least squares criterion is considered, the objective function can also be composed.

(12) $$\begin{array}{c} {\tilde{\phi }}_{1}\left(x\right)={\left(P-R\overset{\wedge }{x}\right)}^{T}\left(P-R\overset{\wedge }{x}\right)+x^{T}\left(B+I\right)x, \end{array}$$

where minimum $\hat{X}$, ${\tilde{\phi }}_{1}\left(\hat{x}\right)$ are selected so that the least squares criterion, maximum uniformity criterion, and smoothness criterion are integrated. Iterative reconstruction technique can be expressed as below.

(13) $$\begin{array}{c} Ax=p,\\ x=\left[x_{1},x_{2},\ldots ,x_{n}\right]T,\\ p=\left[p_{1},p_{2},\ldots ,p_{m}\right]T, \end{array}$$

*x* is the one-dimensional vector of *n* elements, each element represents the pixel of a 2D object, *p* is the one-dimensional vector of *m* elements, each element represents the measurement carried out by a detector in a projection direction, *A* is called the projection matrix, and each value in this matrix called the projection rate defines the contribution of specific pixels to specific projection values.

**Figure 2:**
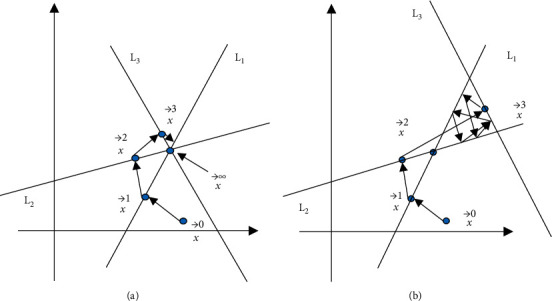
Iterative process. (a) Compatible equation group. (b) Incompatible equation group.

The implementation of iterative reconstruction algorithm is as follows.

(14) $$\begin{array}{c} X^{\left(k+1\right)}=X^{\left(k\right)}+\lambda_{k}\frac{P_{i}-\langle a_{i},x_{k}\rangle }{{\left\|a_{i}\right\|}^{2}}a_{i}^{T}, \end{array}$$

where *λ* is the relaxation factor that determines the convergence rate. The whole iterative process is given in Figure 2.

$\overset{⟶\text{0}}{x}$ is an arbitrary initial value. The first step is to project the point $\overset{⟶\text{0}}{x}$ vertically to L1 to get a new value $\overset{⟶1}{x}$. The next step is to project the point $\overset{⟶1}{x}$ vertically to L2 to get new a value $\overset{⟶2}{x}$, and so on in a similar fashion. Each step is to project the currently estimated point onto the next line so that it satisfies the next equation. Eventually, the algorithm will converge on the solution of the equation group [22].  Figure 2(a) is a compatible equation group; that is, there is an exact solution for the equation group. When the number of iterations is sufficient, a convergence value can be obtained.  Figure 2(b) is a system of incompatible equation group that will jump all the time within the triangular region in the middle after the number of iterations to a certain value. It means that any point in the triangular region is a numerical solution of the equation group, and the better way is to obtain multiple solutions and then calculate their average values.

### 2.5. Evaluation Indicators of Image Quality

The reconstructed image sharpness and noise will vary due to different image processing methods, so the mean square error (MSE) and structural similarity (SSIM) are used to evaluate the processed images.

MSE_out_ represents the MSE between the result obtained by the reconstruction algorithm and the standard result. If the MSE_out_ value is >1, it indicates that the new reconstruction algorithm is better. The greater the value, the better the effect. MSE_in_ represents the *MSE* between the result obtained by the reconstruction algorithm for contrast and the standard result, and the improvement in signal-to-noise ratio (ISNR) is expressed as follows.

(15) $$\begin{array}{c} \text{ISNR}=10\;\log_{10}\left(\frac{{\text{MSE}}_{\text{in}}}{{\text{MSE}}_{\text{out}}}\right). \end{array}$$

*MSE* represents the mean square error between the reconstruction result and the target image, and *μ*^2^max represents the maximum pixel value in the reconstruction result. The peak signal-to-noise ratio (PSNR) can be expressed as follows.

(16) $$\begin{array}{c} \text{PSNR}=10\;\log_{10}\left(\frac{\mu^{2}\;\max}{\text{MSE}}\right). \end{array}$$

*MSE* is the variance between the reconstructed image and the reference image, and *EMSE* equation is expressed as below.

(17) $$\begin{array}{c} \text{EMSE}=\sqrt{\text{MSE}}. \end{array}$$

*SSIM* is a measure of the similarity between images. The closer the value is to 1, the higher the similarity between images is. In image reconstruction, the similarity between the reconstructed image and the original image can be measured. If *a*,*b* represent the results obtained by the new reconstruction method and standard results in the window, respectively, *μ*_*a*_ and *μ*_*b*_ represent the mean corresponding to *a*,*b*, *β*_*a*_ and *β*_*b*_ represent the standard deviation corresponding to *a*,*b*, and *k*_1_ and *k*_2_ are two very small constants so as not to divide by zero. *SSIM* is expressed as follows.

(18) $$\begin{array}{c} E\left(\text{a},b\right)=\frac{\left(2\mu_{\text{a}}\mu_{\text{b}}{+\text{k}}_{1}\right)\left(2\beta_{\text{ab}}{+\text{k}}_{2}\right)}{\left(\mu_{\text{a}}^{2}+\mu_{\text{b}}^{2}{+\text{k}}_{1}\right)\left(\beta_{\text{a}}^{2}+\beta_{\text{b}}^{2}{+\text{k}}_{2}\right)}. \end{array}$$

### 2.6. Statistical Methods

Statistical software SPSS 24.0 was used for statistical processing in this study. The measurement data were expressed as mean ± standard deviation. The measurement data in accordance with normal distribution were compared with single factor and multiple means, and the independent sample *t*-test was used between the two groups. The counting data were expressed as percentage or percentage by chi-square test. *P* < 0.05 was considered as statistically significant difference.

## 3. Results

### 3.1. Low-Dose HRCT Image Reconstruction

**Figure 3:**
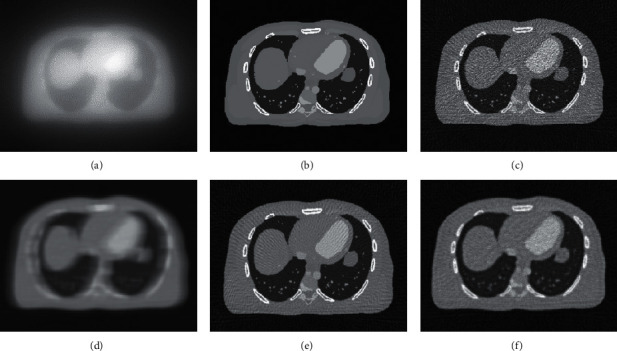
Low-dose HRCT image reconstruction: (a, d) images added with noise; (b) original image; and (e, c, f) reconstructed images.

Figures 3(a) and 3(d) show adding noise through the Poisson distribution and then performing an iterative reconstruction. The image sharpness was very low at this time.  Figure 3(b) is the original HRCT picture, and Figure 3(e) is the figure after one iteration rendering, which is still blurred compared to the original image. Figures 3(c) and 3(f) are the images obtained by reconstructing the projection data based on 90° projection angle using filtered back projection (FBP) and algebraic reconstruction technique (ART), respectively. The reconstructed image was compared with the original image. The image after iterative reconstruction was closer to the original image, and the edge of the image after FBP reconstruction was more blurred and noisier, and the image obtained by iterative reconstruction technique was smoother and clearer.

### 3.2. Comparison of Image Reconstruction Effect

**Figure 4:**
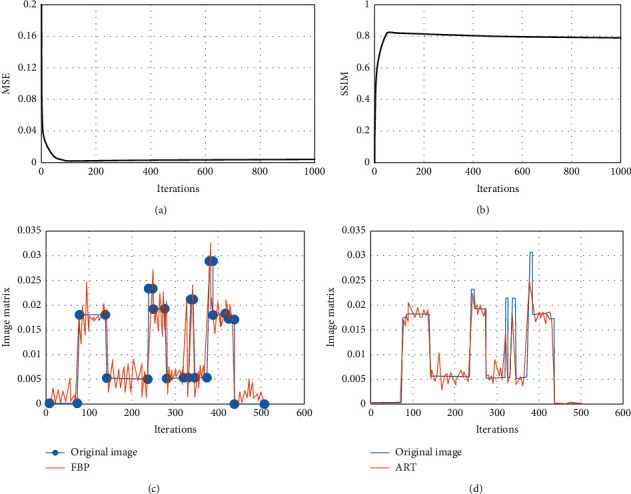
Evaluation of image reconstruction effect. (a), (b) The reconstructed images MSE and SSIM, respectively. (c) The image matrix of original image and FBP image. (d) The image matrix of original image and iterative reconstruction image.

From Figures 4(a) and 4(b), it can be found that after the number of iterations exceeded a certain value, the MSE no longer continues to decrease but begins to grow. It may be because the projection data obtained with only a 90° projection angle, the complexity of the projection data was less than that of the image to be reconstructed. After a certain number of iterations, due to the excess expression ability of the model, some features that can only meet the projection data but cannot meet the original image were trained, which led to the increase of MSE. Figures 4(c) and 4(d) are the contrast curves of FBP, ART, and original images, respectively. The FBP reconstructed image fluctuates greatly, while the ART reconstructed image is more stable. Although the FBP image fluctuated greatly, there was no energy loss. Although the ART curve performs better at the smooth, there is a large gap with the curve of the original image at the peak, which will lead to loss of image details.

### 3.3. Imaging Findings of BO

**Figure 5:**
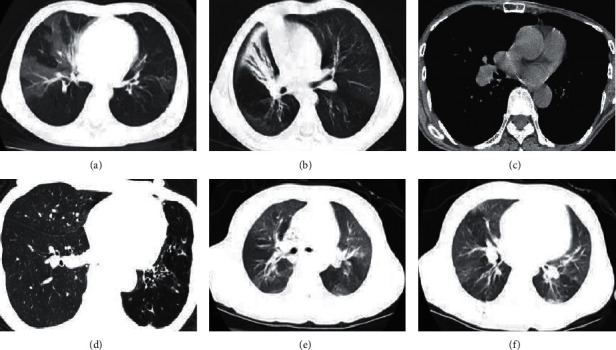
HRCT iterative reconstruction image of BO. (a, b, e, and f) HRCT plain scan images; (c, d) Expiratory phase HRCT images.

HRCT of BO are typically characterized by decreased lung density, accompanied by decreased vascular caliber. The features are called mosaic perfusion or mosaic reduction, suggesting bronchial or bronchiolar air trapping, which can be displayed by HRCT scanning in the expiratory phase. In patients with disease progression, there will be bronchiectasis characterized by airway wall thickening and caliber expansion. From the HRCT images of children after iterative reconstruction in Figure 5, the peripheral bronchiole wall was thickened, bronchiole dilatation was accompanied by secretion retention, and lobular central bronchial nodules were observed. Figures 5(c) and 5(d) show that the left pulmonary artery is smaller than the right pulmonary artery, the volume of the left pulmonary artery is reduced, the pulmonary transparency is enhanced, and the blood vessels are thinner.

### 3.4. Basic Information of Patients

**Figure 6:**
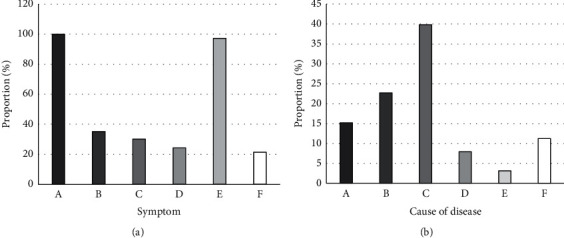
Etiology and symptoms of the patients. In Figure (a), A, B, C, D, E, and F indicated wheezing, shortness of breath, dyspnea, wheezing or rind in the lungs, cough, and cyanosis around the lips, respectively. In Figure (b), A, B, C, D, E, and F represented mycoplasma *pneumoniae*, adenovirus with mycoplasma *pneumoniae*, adenovirus, influenza virus, parainfluenza virus, and other causes, respectively.

Table 1 showed the basic information, such as age and course of disease, of the children included in the study. The children aged 8–11 accounted for a relatively high proportion (38.79%). Most of the patients (46.8%) had a course of disease from five to ten months. There was no statistical significance in patients' basic information (*P* > 0.05).  Figure 6 suggests the analysis results of the main causes and symptoms of the children. All the children included in the study had wheezing symptoms, and more than 97% of the children had cough symptoms. The other common symptoms were shortness of breath, dyspnea, and so on, mainly including adenovirus, mycoplasma *pneumoniae*, and influenza virus.

### 3.5. Improvement of Pulmonary Function Obstruction

**Figure 7:**
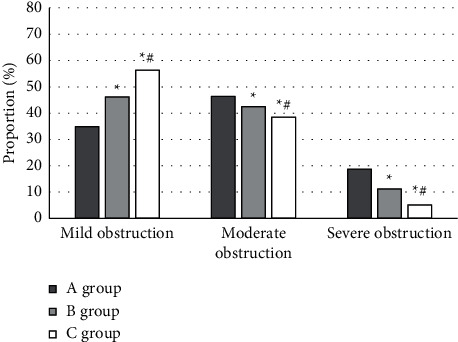
Degree of pulmonary function obstruction in patients. ^*∗*^Compared to group A (*P* < 0.05). ^#^Compared to group B (*P* < 0.05).

Children with BO are generally accompanied by obstructive ventilatory dysfunction mainly with small airway involvement, which can be divided into mild obstruction, moderate obstruction, and severe obstruction according to the degree of obstruction.  Figure 7 suggests the comparison results of the degree of pulmonary function obstruction among the three groups after different ways of guided TCM treatment. The proportion of patients with moderate-to-severe obstruction in group C was 5.18%, obviously inferior than that in group A (18.75%) and group B (11.29%), and significantly lower in group B than that in group A (*P* < 0.05). The proportion of patients with mild obstruction in group C was 56.38%, which was significantly superior than that in group A (34.93%) and group B (46.21%), and the proportion in group B was significantly higher than that in group A (*P* < 0.05).

### 3.6. Clinical Efficacy Analysis

**Figure 8:**
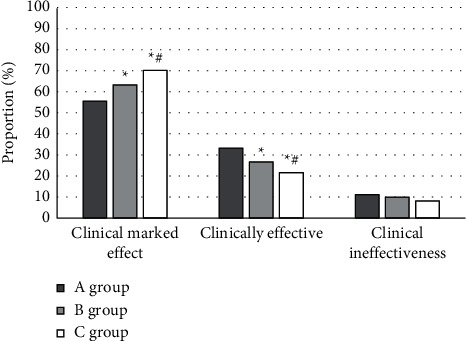
Analysis of clinical efficacy in patients. ^*∗*^Compared to group A (*P* < 0.05). ^#^Compared to group B (*P* < 0.05).

After the three groups of patients were treated with TCM guided by different ways, the efficacy was evaluated according to the improvement of patients' symptoms and expressed by three levels: clinically significantly effective, clinically effective, and clinically ineffective.  Figure 8 reveals the efficacy comparison results of the three groups of patients. The proportion of clinically significant effect after treatment in group C was 70.18%, which was clearly higher than that in group A (55.5%) and group B (63.34%), and the proportion in group B was clearly higher than that in group A (*P* < 0.05).

## 4. Discussion

At present, the treatment of bronchiolitis obliterans in children is mainly treated with glucocorticoids and bronchodilators, and the key stage of clinical treatment of this disease is the early stage. Therefore, accurate and early diagnosis also plays a key role in its efficacy. Katsura et al. [23] found that the application of TCM therapy can significantly improve the clinical symptoms and pulmonary ventilation function of children. In addition, TCM therapy has less impact and side effects on children, so it has good application value and research prospects [24]. Early chest radiographs of patients with BO are often unremarkable, and pulmonary hyperinflation and linear shadows and reticular shadows indicating alveolar wall thickening lack specificity for their diagnosis. Without contrast agent, HRCT plain scan at the end of inspiratory and end of exhalation is an effective method for noninvasive diagnosis of BO. Before and at the early stage of the clinical manifestations of BO, HRCT mosaic perfusion sign cannot be used as the basis for its early diagnosis. However, if the children are accompanied by abnormal changes in the bronchus (e.g., thickening of the bronchial wall and gas retention in the expiratory phase), it has a key role in prompting the diagnosis of BO.

The results showed that the HRCT image of lung after iterative reconstruction was closer to the original image than the filtered back projection reconstruction. The edge of the image reconstructed by filtered back projection was more blurred and the noise was greater. The image obtained by iterative reconstruction technique was smoother and clearer. The image stability after iterative reconstruction was higher. HRCT of the lung after iterative reconstruction can display the lesion site more clearly and intuitively, which has a key guiding role in the early diagnosis and treatment of bronchiolitis obliterans. After treatment, the proportion of patients with moderate and severe obstruction in group C was significantly lower than that in group A and group B, and that in group B was significantly lower than that in group A (*P* < 0.05). The proportion of patients with mild obstruction in group C was significantly higher than that in groups A and B, and that in group B was significantly higher than that in group A (*P* < 0.05), which was similar to the results of Weng et al. [25]. Under the guidance of HRCT images after iterative reconstruction, a targeted TCM treatment plan for eliminating phlegm and removing blood stasis was developed, which had better efficacy for children with bronchiolitis obliterans and significantly improved their lung ventilation function. The proportion of clinical effect in group C was significantly higher than that in group A and group B (*P* < 0.05). The TCM therapy of resolving phlegm and removing blood stasis showed good efficacy and safety in improving the symptoms, signs, and pulmonary ventilation function of children with bronchiolitis obliterans. Moreover, guided by the HRCT images after iterative reconstruction, the targeted formulation of TCM treatment plan for resolving phlegm and removing blood stasis further improved its comprehensive efficacy.

## 5. Conclusion

The results showed that the HRCT of the lung after iterative reconstruction could display the lesion site more clearly and intuitively, which played a key guiding role in the early diagnosis and treatment of bronchiolitis obliterans. HRCT image based on iterative reconstruction technology combined with TCM therapy to remove blood stasis and remove phlegm had a good therapeutic effect on children with high application value. However, the HRCT image processing in this study lacks comparison with other intelligent algorithms, and the samples included in the study are few and are of low representativeness. Therefore, these aspects will be improved and optimized in the subsequent experiments, and the application of HRCT images based on artificial intelligence algorithm in the diagnosis and treatment of bronchiolitis obliterans in children will be further studied. In conclusion, this study provides a reference for the early diagnosis and treatment of bronchiolitis obliterans in children.

## Figures and Tables

**Table 1:** Basic information of patients.

Item	Category	Proportion (%)
Age (years)	4∼7	25.33
	8∼11	38.79
	12∼15	35.88

Gender	Male	53.33
	Female	46.67

Course of disease (month)	<5	33.4
	5∼10	46.8
	>10	19.8

## Data Availability

The data used to support the findings of this study are available from the corresponding author upon request.
